# Editorial: Implications of lipids and modified lipoproteins in atherogenesis: from mechanisms towards novel diagnostic and therapeutic targets

**DOI:** 10.3389/fcvm.2023.1245716

**Published:** 2023-07-24

**Authors:** Simon Kraler, Tatsuya Sawamura, Grace Yen-Shin Harn, Chu-Huang Chen, Alexander Akhmedov

**Affiliations:** ^1^ Center for Molecular Cardiology, University of Zurich, Schlieren, Switzerland; ^2^ Department of Molecular Pathophysiology, Shinshu University School of Medicine, Shinshu University, Matsumoto, Japan; ^3^ HEART, Health Resource Technology, LLC, Houston, TX, United States; ^4^ Vascular and Medical Research, The Texas Heart Institute, Houston, TX, United States

**Keywords:** low-density lipoprotein, inflammation, biomarkers, risk stratification, residual cardiovascular risks, high-density lipoproteins, triglycerides

**Editorial on the Research Topic** Implications of lipids and modified lipoproteins in atherogenesis: from mechanisms towards novel diagnostic and therapeutic targets

## 1. Introduction

Atherosclerosis is the leading cause of morbidity and mortality worldwide. Despite the unprecedented gains over the past decades, patients with established atherosclerotic cardiovascular disease (ASCVD) remain at high risk of recurrent ischemic events despite optimal management (1–3). Deepening insights into the underlying mechanisms provide unique opportunities to refine previous concepts of atherosclerosis pathobiology with the ultimate goal to improve its prevention and treatment and eventually patient outcomes (4). The atherogenicity of apolipoprotein-B containing lipoproteins is well established, but recent studies have shed new light on the importance of lipid quality in the development of atherosclerosis and its clinical complications (5, 6).

With this Research Topic, we have assembled an issue of compelling articles, comprising original research, case reports, editorials, and state-of-the-art reviews, with the aim to give the readers of *Frontiers in Cardiovascular Medicine* a comprehensive overview of the role of modified lipoproteins in the different phases of atherogenesis. Indeed, this Research Topic not only deepens our understanding of lipid-driven mechanisms underpinning ASCVD but also provides insights into novel concepts to address the high burden of ASCVD.

## 2. Low-density lipoproteins, high-density lipoproteins, and triglycerides

With the accumulation of evidence on sex-specific differences in atherosclerosis pathobiology, clinical tools to guide sex-specific patient care are gaining increasing attention in recent years (7). The narrative review article by Wang and He highlights specific differences in lipoprotein metabolism and associated risk factors in women and men. For example, women tend to have higher high-density lipoprotein cholesterol (HDL-C) levels, which are observationally linked to lower ASCVD risk; however, at the same time, women tend to have higher triglyceride levels, which are associated with higher cardiovascular risk. Furthermore, sex hormones and reproductive factors may affect lipoprotein metabolism and, thus, the risk of major adverse cardiovascular events (MACE). Gaining a better understanding of sex-specific differences may open novel avenues for clinical interventions that could improve the prevention and treatment of ASCVD.

Taking this concept a step further, Dietrich et al. have emphasized the importance of sex hormones and sex-specific effects of HDL and its interaction with endothelial cells. In addition to their immunomodulatory, anti-inflammatory, and anti-oxidative properties, HDL particles are thought to provide vasculoprotective effects by promoting vasorelaxation and regulating vascular lipid metabolism. An improved understanding of how sex-specific factors affect these interactions may be useful in developing personalized approaches for preventing and treating ASCVD.

In the review article by Tirandi et al. the potential role of physical activity in regulating the expression of proprotein convertase subtilisin/kexin type 9 (PCSK9), a key regulator of LDL-C levels, is discussed. In a related study, Wang et al. investigated potential associations between PCSK9 levels and platelet reactivity in individuals not receiving statin or antiplatelet therapy. The authors reported a significant positive correlation between PCSK9 and platelet reactivity, suggesting that inhibiting PCSK9 may attenuate platelet reactivity and thus MACE risk in patients at high ASCVD risk. This discovery encourages further research on the pleiotropic effects of PCSK9 inhibition in caring for patients with established ASCVD.

The retrospective study by Wang and He assessed the correlation between cardiometabolic risk factors and obesity in 103 patients with familial hypercholesterolemia. Their findings demonstrate the potential of using novel biomarkers for risk stratification and personalized management of moderate to high-risk patients, such as those with familial hypercholesterolemia. Similarly, the study by Zeng et al. emphasizes the importance of considering non-HDL-C as a risk factor in men not receiving statin therapy. The authors describe a U-shaped relationship between non-HDL-C levels and all-cause and cardiovascular mortality, suggesting that both low and high levels of non-HDL-C are associated with increased mortality risk, a finding that was similarly reported for LDL-C (8).

Xu et al.'s study focused on comparing established formulas used for calculating LDL-C levels in fasting and postprandial states in the Chinese population. Notably, the Friedewald formula provided the highest accuracy for determining fasting LDL-C levels, whereas the Sampson formula performed better for measuring postprandial LDL-C levels. These findings may stimulate further research in this direction to accurately assess cardiovascular risk and eventually refine interventions in the Chinese population.

In an important review on macrophage-mediated pinocytotic engulfment of lipoproteins, Miyazaki highlights a receptor-independent endocytic pathway for foam cell formation. This process appears to occur when lipoproteins accumulate around inflammatory cells and involves plasma membrane ruffling, small GTPases, and cytoskeletal rearrangement. Although native LDL may not be the main driver of foam cell formation, further experimental studies are necessary to identify the master regulator of lipoprotein engulfment by macrophages to improve our understanding of its role in ASCVD pathogenesis.

## 3. Quality of lipoproteins

One area of research that has gained increasing attention in recent years is the role of modified lipoproteins in the life cycle of atherosclerotic plaque. Modified lipids include oxidized low-density lipoprotein (oxLDL), small-dense LDL, triglyceride-rich LDL, electronegative LDL, and very low-density lipoprotein (VLDL) (5, 9–11).

Lee et al.'s review focuses on VLDL, a potential driver of cardiometabolic diseases. The most electronegative VLDL subclass exerts cytotoxic effects on the endothelium and has been linked to chronic coronary syndromes and atrial remodeling in patients with metabolic syndrome. The review article highlights the significance of postprandial VLDL modification and the need for further investigation into the role of VLDL subclasses in the pathobiology of cardiometabolic diseases.

In a Bayesian network analysis focused on biomarkers of coronary atherosclerosis, Voros et al. have highlighted the importance of triglyceride-rich LDL particles in ASCVD development. In the 665 patients included in the analysis, LDL-triglycerides were directly linked to carotid atherosclerosis in over 95% of the models. Interestingly, genetic variants in the LIPC gene (encoding hepatic lipase) were associated with LDL-triglyceride levels and the presence of atherosclerotic plaque. These findings suggest that triglyceride-rich LDL particles may play a crucial role in atherosclerosis development, thus providing a potential target for future studies.

In a Chinese population, Liu et al. explored potential associations between remnant cholesterol (RC) and new-onset carotid plaques. The authors concluded that elevated RC levels are strongly associated with the presence of new-onset carotid plaques relative to other lipid parameters, particularly in those with low LDL-C levels. This study highlights the importance of considering RC in predicting residual cardiovascular risk, such as in those with low levels of LDL-C. A meta-analysis by Tian et al. further evaluated the prognostic value of RC in patients with chronic coronary syndromes. They reported an increasing risk of MACE with higher RC levels, although no significant association with all-cause mortality was identified.

The review article by Shen et al. provides insights into the impact of dyslipidemia on coronary collateral formation during diabetic states. The authors note that both altered serum lipid profiles and glycoxidative modification of lipoproteins contribute to endothelial dysfunction and blunt endothelial progenitor cell responses and interfere with the growth and maturation of collateral vessels in diabetic patients with chronic coronary syndromes.

The above review article and the aforementioned work demonstrate the significance of lipids in atherosclerosis and its acute and chronic clinical sequelae (Miyazaki, Voros et al. Shen et al.). Further research in this area may lead to innovative strategies for preventing and treating ASCVD and ultimately improving patient outcomes.

Hong et al. conducted a systematic review and meta-analysis to evaluate the relationship between oxLDL and cardiovascular disease in patients with chronic inflammatory conditions. The analysis of three observational studies comprising a total of 1,060 participants showed that circulating oxLDL levels are increased in participants with ASCVD during chronic inflammatory conditions. Indeed, their study suggests that oxLDL may be a useful biomarker for risk stratification of patients with established cardiovascular disease driven by chronic inflammation.

Since the introduction of the traditional risk factor concept in the Framingham Study (12), cholesterol-rich lipoproteins have been extensively examined, with instrumental variable approaches now allowing for causal inference using observational data. In the innovative study by Jin et al., the causal role of cholesterol efflux capacity in chronic coronary syndromes, acute myocardial infarction, and ischemic stroke was examined using Mendelian randomization. Considering the potential limitations of their approach, these findings suggest that increased cholesterol efflux capacity reduces the risk of chronic coronary syndromes and myocardial infarction but has a weaker causal effect on ischemic stroke, likely because of its more heterogeneous pathobiology.

Collectively, the studies by Wang et al. Zeng et al. Xu et al. Liu et al. Tian et al. and Jin et al. highlight the importance of considering multiple factors in assessing cardiovascular risk with potential future therapeutic implications. In addition to traditional risk factors, non-HDL-C and RC concentrations may provide valuable insights into a patient's overall cardiovascular risk.

These studies also highlight the interplay of lipoproteins, physical activity, sex-specific determinants, and emerging biomarkers in cardiovascular health and disease (Wang and He). Although the traditional risk factor concept has long prevailed, it might have resulted in an oversimplified understanding of ASCVD. An improved understanding of ASCVD pathobiology offers unique opportunities to further improve patient care and to mitigate residual cardiovascular risk.

In their review article, Durrington et al. highlight a critical role of HDL-contained serum paraoxonase-1 (PON1) in protecting against harmful LDL oxidation, a mechanism that drives early phases of ASCVD. Accordingly, reduced serum PON1 activity is associated with dyslipidemic, diabetic, and inflammatory states. Low PON1 levels are further linked to adverse cardiovascular events, particularly in patients with diabetes and established ASCVD, providing a conceptual framework to study functional determinants of HDL to reduce residual cardiovascular risk.

The articles by Wang and He, Dietrich et al., Tirandi et al., Lee et al., Hong et al., and Durrington et al. underscore the significance of biomarkers in cardiovascular disease prevention and personalized patient care. The identification of novel biomarkers can aid in personalized risk assessment and provide a basis for targeted interventions in high-risk patients. Moreover, these studies suggest that novel biomarkers, including oxLDL, PON1, and electronegative VLDL, may serve as complementary tools for the identification of patients at particularly high ASCVD risk who may benefit from intensive secondary prevention measures.

However, it is essential to recognize that these biomarkers should not be used in isolation but should be considered alongside other clinical tools, including risk scores. Moreover, the clinical utility of novel biomarkers for risk assessment must undergo rigorous validation in different populations and settings, taking into account potential limitations inherent in the design of the studies noted above.

**Figure 1:**
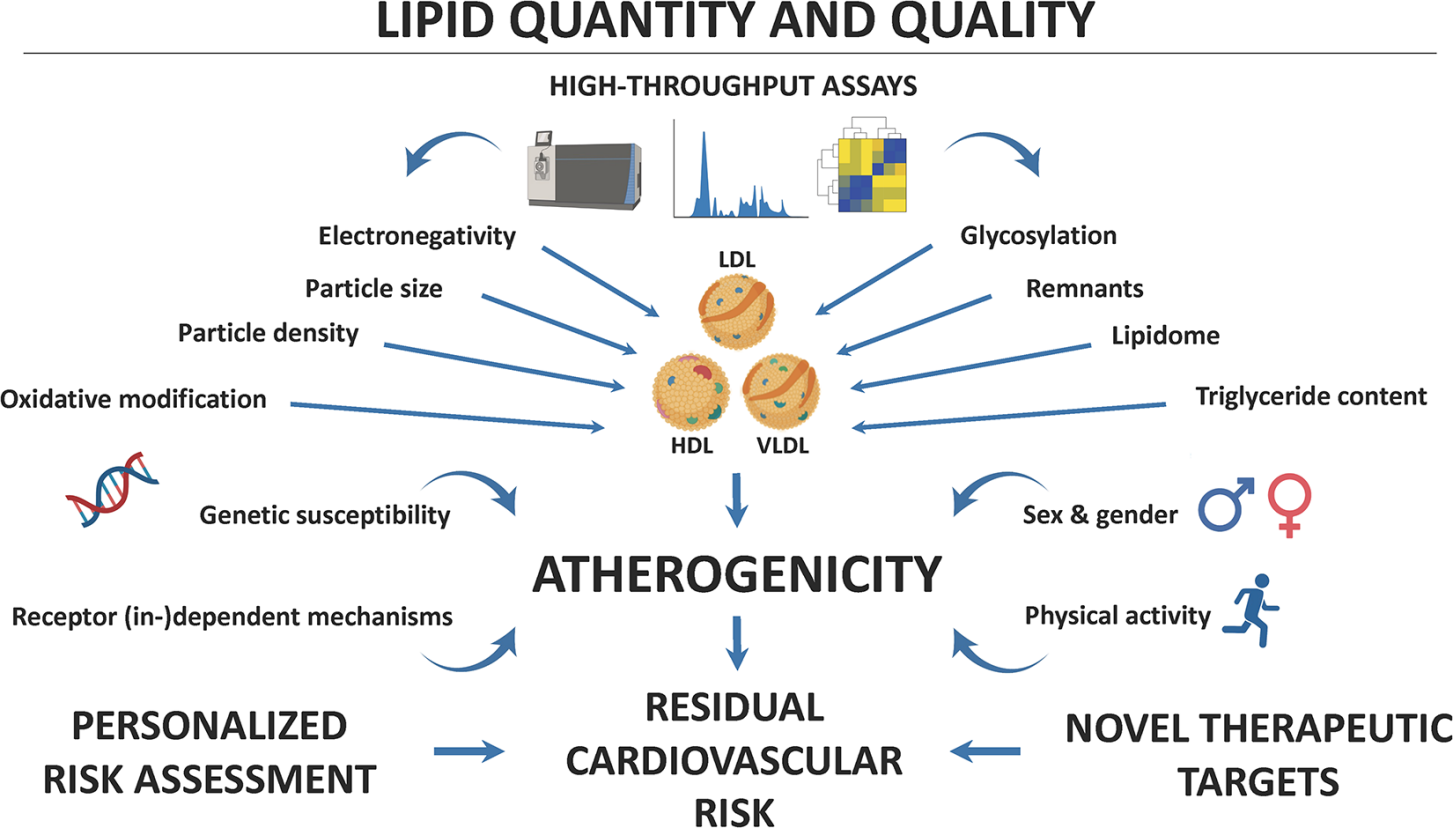
Lipids and their modifications contribute importantly to the pathogenesis of atherosclerotic cardiovascular disease (ASCVD). High-throughput technologies, including lipidomics, now allow the in-depth characterization of lipoprotein structure and function. Both physical and chemical characteristics modulate the atherogenic effects of blood lipids, but genetic susceptibility, sex and gender, physical activity, and receptor (in-)dependent mechanisms can determine their (cardio-)vascular effects. A better understanding of how these biomarkers contribute to ASCVD pathogenesis may allow for personalized risk assessment and the identification of novel therapeutic targets, aimed at reducing residual cardiovascular risk and thus improving outcomes of patients at particularly high cardiovascular risk.

Together, the studies presented in this Research Topic highlight the importance of biomarkers in the prevention and management of ASCVD and its complications (Figure 1). The identification and rigorous validation of novel biomarkers could assist in personalized risk assessment to guide targeted interventions in high-risk populations. To that end, high-throughput assays are needed for qualitatively assessing changes in the structure and function of lipoproteins to work toward the clinical implementation of multidimensional lipid profiling. The herein proposed research pursuit is crucial for developing effective prevention and management strategies for patients at risk for or with established ASCVD with the ultimate goal of reducing the burden of residual cardiovascular risk.
